# Trends in Treatment Need and Receipt for Substance Use Disorders in the US

**DOI:** 10.1001/jamanetworkopen.2024.53317

**Published:** 2025-01-06

**Authors:** Ligang Liu, Chen Zhang, Milap C. Nahata

**Affiliations:** 1 Institute of Therapeutic Innovations and Outcomes, College of Pharmacy, The Ohio State University, Columbus; 2 University of Nebraska Medical Center, Omaha, Nebraska; 3 College of Medicine, The Ohio State University, Columbus

## Abstract

This cross-sectional study uses data from the National Survey on Drug Use and Health (NSDUH) to investigate trends in substance use disorder treatment need and receipt from 2013 to 2023 among the US population.

## Introduction

Despite the profound consequences of substance use disorder (SUD), treatment rates have remained critically low.^1^ The COVID-19 pandemic exacerbated these issues by disrupting health care services and increasing stress and isolation.^2^ This study aimed to describe treatment needs, treatment receipts, perceived needs, and treatment barriers during 2013 to 2023.

## Methods

This cross-sectional study was deemed exempt from additional institutional review board review by The Ohio State University because of the use of deidentified data. Informed consent was obtained from all participants. This study is reported following the STROBE reporting guideline. We analyzed National Survey on Drug Use and Health (NSDUH) annual reports in August 2024, which provided continuous cross-sectional data on SUD among civilian, noninstitutionalized populations aged 12 years and older in the US. Respondents were classified as needing treatment if they met *Diagnostic and Statistical Manual of Mental Disorders* (Fifth Edition) criteria. SUD were categorized into alcohol use disorder (AUD) and drug use disorder (DUD) defined by NSDUH, with opioid use disorder (OUD) highlighted owing to its enormous impact. OUD prevalence data were available from 2016, and treatment data became available in 2019. Participants were asked if they perceived an unmet need for treatment, and those who indicated unmet needs were further asked to specify reasons for not receiving care. Summary statistics, including point estimates and 95% CIs, were obtained directly from annual reports. This study is descriptive in nature and we did not test for statistical significance.

## Results

**Figure 1.**
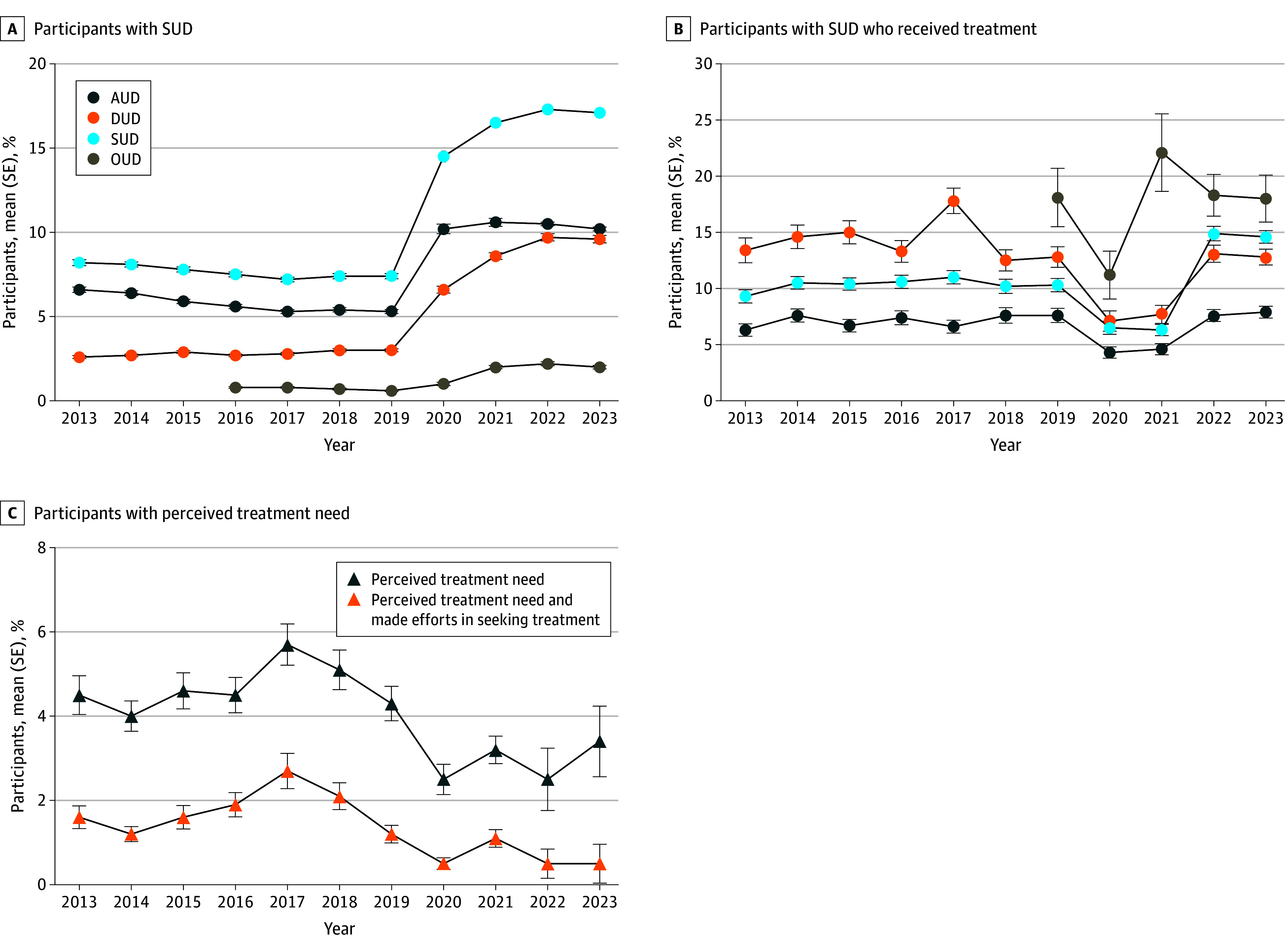
Prevalence of Substance Use Disorder (SUD) The figure shows the percentage of participants classified as needing substance use treatment among the general population aged 12 years or older (A), percentage of participants with SUD who received treatment (B), and percentage of patients with perceived treatment need overall and the percentage of among them who made efforts in seeking treatment (C). All data are presented as means and standard errors (SEs). SUD includes alcohol use disorder (AUD) and drug use disorder (DUD), which was defined as meeting *Diagnostic and Statistical Manual of Mental Disorders* (Fifth Edition) SUD criteria for 1 or more of the following drugs: marijuana, cocaine, heroin, hallucinogens, inhalants, methamphetamine, or prescription psychotherapeutic drugs (ie, pain relievers, stimulants, and tranquilizers or sedatives). OUD indicates opioid use disorder and is highlighted owing to its enormous impact.

Among 657 583 participants, the prevalence of individuals needing SUD treatment increased from 8.2% in 2013 to 17.1% in 2023. AUD increased from 6.6% to 10.2%, while DUD increased from 2.6% to 9.6%. OUD more than doubled from 0.8% in 2016 to 2.0% in 2023 (Figure 1A). Despite increasing treatment needs, the percentage of participants receiving treatment decreased from 9.3% in 2013 to 6.5% in 2020. However, SUD treatment rates rebounded to 14.9% in 2022 and stabilized thereafter. AUD treatment decreased from 6.3% in 2013 to 4.3% in 2020, recovering to 7.6% in 2022. Similarly, DUD treatment declined from 13.4% in 2013 to 7.1% in 2020 but rebounded to 13.1% in 2022. OUD treatment decreased to 11.2% in 2020, rebounding to 22.1% in 2021 and decreasing to 18.3% in 2022 (Figure 1B).

**Figure 2.**
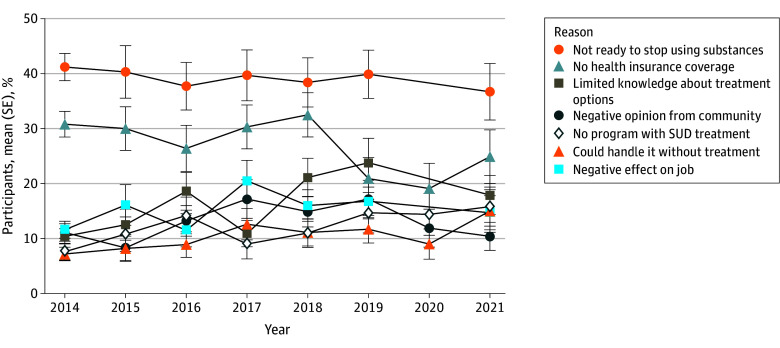
Reasons for Not Receiving Treatment The figure shows the main reasons for not receiving treatment among persons who felt a need but did not receive treatment. All data are presented as means and standard errors (SEs). SUD indicates substance use disorder.

Over the decade and among all participants with SUD who did not receive treatment, 5.7% had a perceived need for treatment and 2.7% perceived a need for treatment and made efforts in seeking treatment (Figure 1C). These numbers deteriorated further after 2020. The main reasons for not receiving treatment included reluctance to stop substance use, insufficient health care coverage, lack of awareness about treatment programs, and negative impacts on employment and community perceptions (Figure 2).

## Discussion

This cross-sectional study’s analysis underscores a public health crisis of SUD. The prevalence of SUD surged during the COVID-19 pandemic, yet the receipt of treatment declined initially as health care services were disrupted. Treatment rates began to recover in 2022, likely due to reopened treatment programs and increased telehealth use.^3^ Alarmingly, our findings revealed that less than 3% of individuals perceived a need for treatment and sought care among those who required but did not receive care, with this number decreasing even further after 2020.

These results call for urgent interventions to bridge the gap between the need for and receipt of SUD treatment, especially after the pandemic. Effective strategies should include enhancing access to counseling services, expanding insurance coverage for treatments, raising public awareness through targeted campaigns, and integrating SUD care into primary health care settings.^4,5,6^ This call to action is vital for mitigating ongoing consequences of SUD and should be a priority of public health services. One study limitation was the reliance on self-reported data.

## Notes

Supplement.
Data Sharing Statement
