# Supplementary material for: Trends in Treatment Need and Receipt for Substance Use Disorders in the US
Source: JAMA Netw Open. 2025 Jan 6;8(1):e2453317. doi: 10.1001/jamanetworkopen.2024.53317 (PMC11704973; doi:10.1001/jamanetworkopen.2024.53317)
Supplement: Supplement. — Data Sharing Statement [file jamanetwopen-e2453317-s001.pdf]

Data Sharing Statement

Liu. Trends in Treatment Need and Receipt for Substance Use Disorders in the US. JAMA Netw Open. Published online January 6, 2025. doi:10.1001/jamanetworkopen.2024.53317

Data
Data available: No
